# Retraction: p53 promotes ZDHHC1-mediated IFITM3 palmitoylation to inhibit Japanese encephalitis virus replication

**DOI:** 10.1371/journal.ppat.1012698

**Published:** 2024-11-08

**Authors:** The PLOS Pathogens Editors

After this article [1] was published, concerns were raised regarding Figs 2–4.

Upon editorial follow-up, a concern about similarities between the β-actin panels in Fig 3B was considered resolved as the underlying images support that the panels are from distinct samples. However, several concerns could not be fully resolved.

Specifically:

- The IFITM3 panel in Fig 2C is a duplicate of lanes 2–5 of the p53 panel in Fig 2E with altered aspect ratio, despite representing different experimental conditions. Additionally, the β-actin panel in Fig 2C is a duplicate of lanes 3–6 of the β-actin panel in Fig 2E with altered aspect ratio. Upon editorial follow-up, the corresponding author indicated there had been an error during data consolidation. The original images underlying Fig 2C were not provided, but western blot images for two sets of replicate experiments conducted at a later date were provided.
- In Fig 3C, when levels are adjusted to visualize the background, there appear to be discontinuities in the background around the band in lane 5 of the 2-BP—IFITM3 panel; in the underlying blot provided, the band in lane 5 appears different to the corresponding band in the published panel. The corresponding author stated that an error was made during the preparation of Fig 3C, but also stated that the original, unadjusted underlying data supports the conclusion presented in [1].
- In editorial assessment of the underlying data provided for Fig 4D, it was noted that the ZDHHC1 and p53 bands do not originate from the same lanes as the corresponding loading controls. The authors stated that the bands for ZDHHC1 and β-actin were selected based on the absence of air bubbles and smears from one of two sets of replicates run on the same blot. Additionally, it was noted that the β-actin bands in the underlying blots did not appear to match those presented in the published figure.

While some original underlying images are available, the corresponding author stated that underlying image data are no longer available for Figs 1A, 1C, 2C, 3E-3F, 5F-5H, S1A, and S7. The available quantitative data were provided on request but did not resolve any of the concerns.

In light of the above issues that raise concerns about the reliability and integrity of the reported data, the *PLOS Pathogens* Editors retract this article.

BL agreed with the retraction. XW, ZW, YL, YY, CX, XL, XX, JWei, DS, KL, XD, JWu, YQ, and ZM either did not respond directly or could not be reached.
